# Long Cephalomedullary Nails Can Be a Cheap and Effective Interval Revision Prosthesis in Infected Hip Replacements That Require Proximal Femoral Replacement: A Small Case Series

**DOI:** 10.1016/j.artd.2023.101218

**Published:** 2023-10-03

**Authors:** Boopalan Ramasamy, Aaron Scott Hammat, Renjy Nelson, Stuart Adam Callary, Ping Keung Chan, Lucian Bogdan Solomon

**Affiliations:** a Department of Orthopaedics and Trauma, Royal Adelaide Hospital, Adelaide, South Australia, Australia; b Centre for Orthopaedic and Trauma Research, The University of Adelaide, Adelaide, South Australia, Australia; c Department of Infectious Diseases, Royal Adelaide Hospital, Adelaide, South Australia, Australia; d Faculty of Health and Medical Sciences, University of Adelaide, Adelaide, South Australia, Australia; e Department of Orthopaedics and Traumatology, Queen Mary Hospital, The University of Hong Kong, Pok Fu Lam, Hong Kong

**Keywords:** Gamma nail, First stage revision, PJI, Temporary spacer, Infected arthroplasty, Cephallomedullary nail

## Abstract

Hip prosthetic joint infection management is complex and expensive, especially in severe bone loss. Reducing the price of interval prosthesis when performing staged revision could minimize costs without compromising outcomes. We present 2 similar techniques developed independently that use an antibiotic-coated cephalomedullary nail with a total hip arthroplasty bearing (head and cemented acetabular component) attached to it as an interval proximal femoral replacement prosthesis. Using this technique, the femoral implant cost was reduced up to 10-fold. All patients have recovered well with resolution of infection and functional recovery similar to patients undergoing proximal femoral replacement. In one case, the lag screw (femoral neck) fractured at 5 months prompting the second-stage revision. This complication should be considered when deciding the timing of second-stage revisions in these cases.

## Introduction

The prevalence of total hip arthroplasty (THA) prosthetic joint infection (PJI) is increasing [1]. In addition to the devastating consequences of PJI on the patient’s health and outcomes, their treatment is costly [1,2]. Treatment of PJI costs triple that of a primary joint arthroplasty and quadruples the patient's length of stay [3]. Chronic PJI requires revision surgery to cure the infection, most often performed as a staged procedure. Several commercial antibiotic-loaded stem spacers are available for the first-stage revision when the proximal femur is preserved [4]. However, reconstruction options are limited when a significant part of the proximal femur was previously destroyed or is resected at the first-stage revision.

In the past, we have treated such cases using an antibiotic-coated proximal femoral replacement (PFR) at first-stage surgery. As more PJI cases are referred to specialized centers, we have seen an increase in case numbers of patients with THA PJI that either require or have already undergone a PFR. Considering the high cost of treatment of PJI, every effort should be made to lower this cost without compromising outcomes. Two years ago, we were referred an obese, immunocompromised patient with severe rheumatoid arthritis and a polymicrobial PJI that had failed several staged revisions, the last of which was a PFR; we planned a repeat staged revision. To better define what antibiotics to use, we planned the first stage as a staged procedure 2 weeks apart to allow for new deep tissue culture results after a period of antibiotic holidays and 2 radical debridements. To avoid a flail limb between the removal of one megaprosthesis and insertion of another and to not use another megaprosthesis for only 2 weeks, we decided to reconstruct the proximal femur using an antibiotic spacer built by coating a Gamma3 nail (Stryker, Schönkirchen, Germany) with antibiotic-loaded cement, to which we added a femoral head at the end of the lag screw and articulated it with a cemented acetabular component. We believed that even if the implant failed during the 2 weeks between planned surgeries, the complication would be minor as we could bring the next operation forward or bedrest the patient for a few days. Surprisingly, the patient did very well and mobilized like our previous patients implanted with a standard PFR prosthesis. Encouraged by the results, we used the technique again in subsequent cases. During expert group discussions, we noted that a similar approach was developed and applied independently in another high-volume revision center in a different continent. Given the procedure’s simplicity, success, and cost savings, we believe it is important to share our experiences.

## Surgical technique

**Figure 1:**
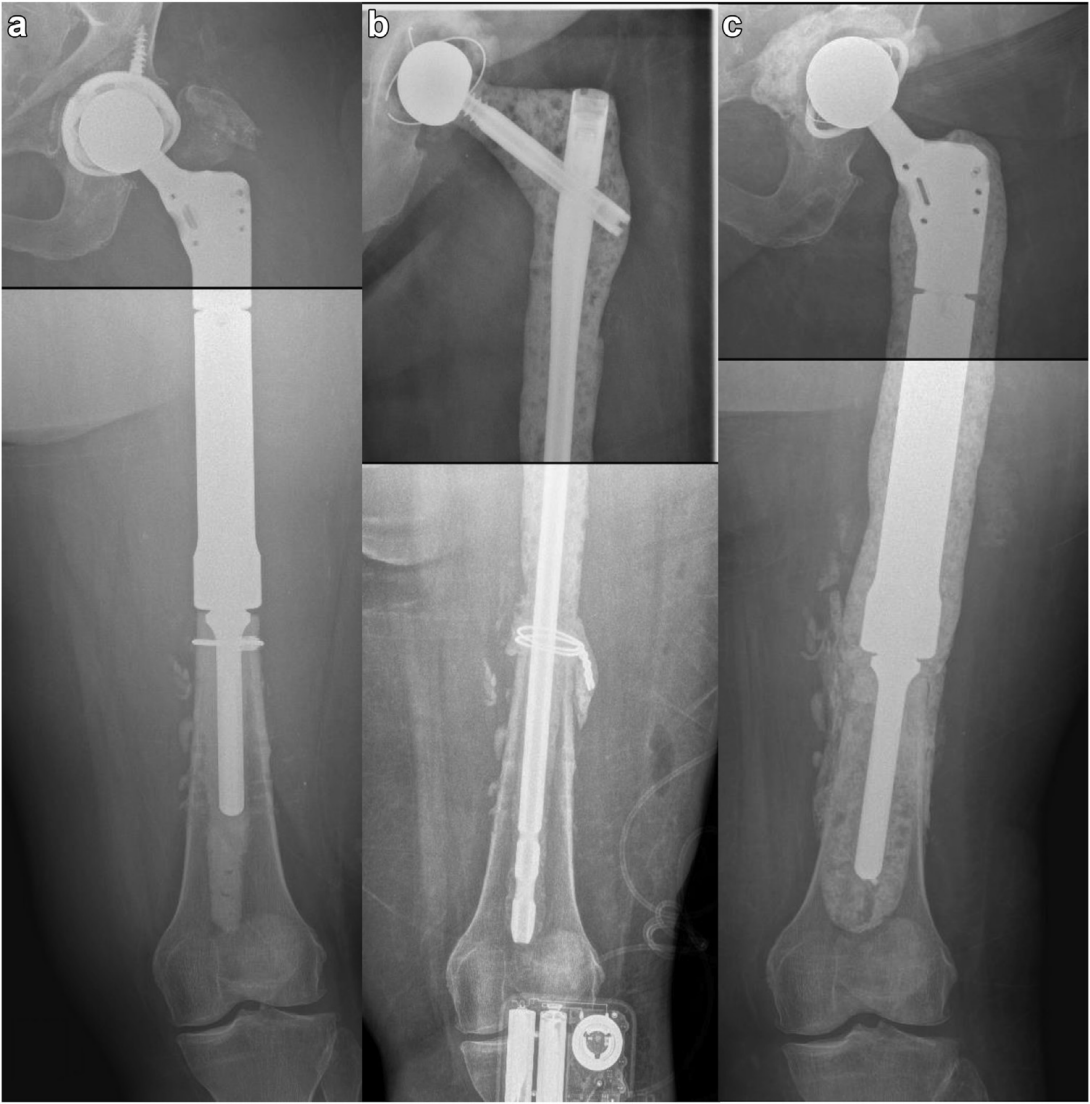
Case 1 RAH. (a) Anteroposterior (AP) radiographs demonstrating infected left proximal femur replacement. (b) AP radiographs after the first-stage reconstruction of the femur using an antibiotic-cement-coated gamma nail as an interval prosthesis. (c) AP radiographs of second-stage revision with an antibiotic-cement-coated proximal femur replacement prosthesis.

**Figure 2:**
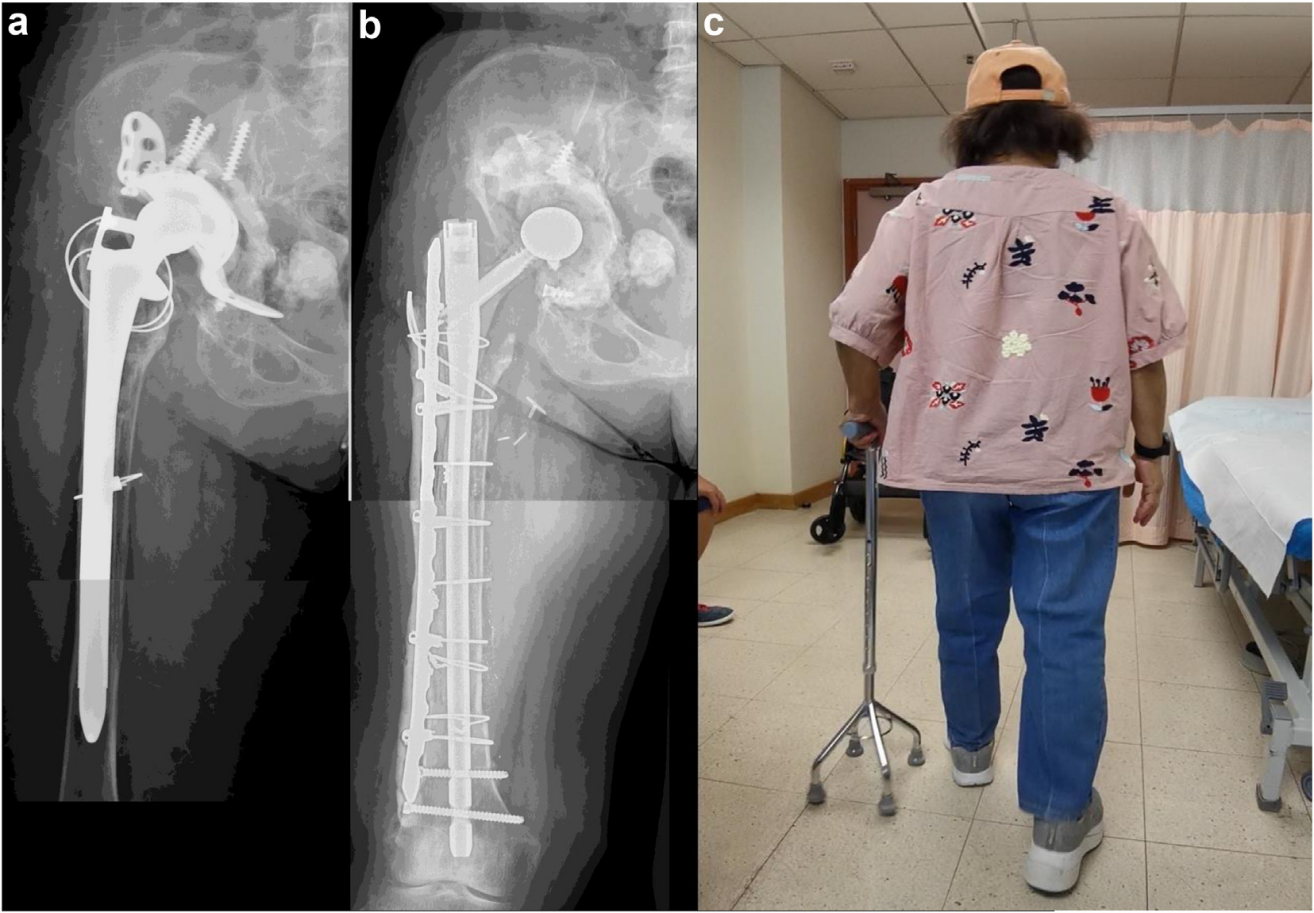
Case1 QMH. (a) AP radiographs demonstrating the failed revision THA with a long cylindrical porous-coated stem in situ. (b) AP radiographs after the first-stage reconstruction of the femur using a gamma nail and lateral femoral plate coated with antibiotic-loaded cement. (c) The patient ambulating in the clinic with a quadpod and a shoe raise 9 months after her first stage revision.

Royal Adelaide Hospital (RAH): The nail and lag screw length is decided on preoperative templating, preferably on a nonoperated contralateral side. We allow 10 cm of the nail to be cemented into the distal femur. The nail is assembled on the back table, and the screw is locked to match the desired length and offset. The nail is then coated with antibiotic-loaded cement, which is applied once the cement starts to wrinkle so that it can be molded by hand. The cement is applied over the entire nail at the desired thickness, ensuring that the cement mantle over the length of the nail inserted in the femoral canal is thinner such that the implant can be introduced and cemented into the remaining femoral canal. Three cement mixes are required to cover the nail and lag screw. A trial femoral head, 12/14 tapper, is used over the top of the lag screw while the cement cures to ensure the desired shape of the proximal nail takes the shape of a femoral stem’s neck. The coated nail is cemented in the femoral shaft using 1 or 2 extra mixes of antibiotic-loaded cement (Fig. 1). At the same time, a small amount of cement is used to cement the desired metal femoral head onto the cement-coated lag screw after removing the trial head. The head is then articulated with a cemented acetabular component from the PROSTALAC system (DePuy/Synthes, Warsaw, IN, USA), implanted as described by Lausmann et al. [5]. Queen Mary Hospital (QMH): The preoperative templating and antibiotic cement coating of the gamma nail were done similarly to the technique above. A mechanically stronger implant was thought to allow for a more extended observation period with the functioning spacer before proceeding to second-stage surgery. To achieve this, the constructs were reinforced with a femoral plate (DePuy/Synthes) (Case 4 and 5), unitized to the gamma nail by multiple cerclage cables applied across the cerclage position pins on the locking screw hole of the plate toward the medial side of the gamma nail as well as locking screws (Fig. 2). Postoperatively, all patients underwent physiotherapy, and they were mobilized for weight bearing as tolerated with a frame for support.

## Cases and results

**Figure 3:**
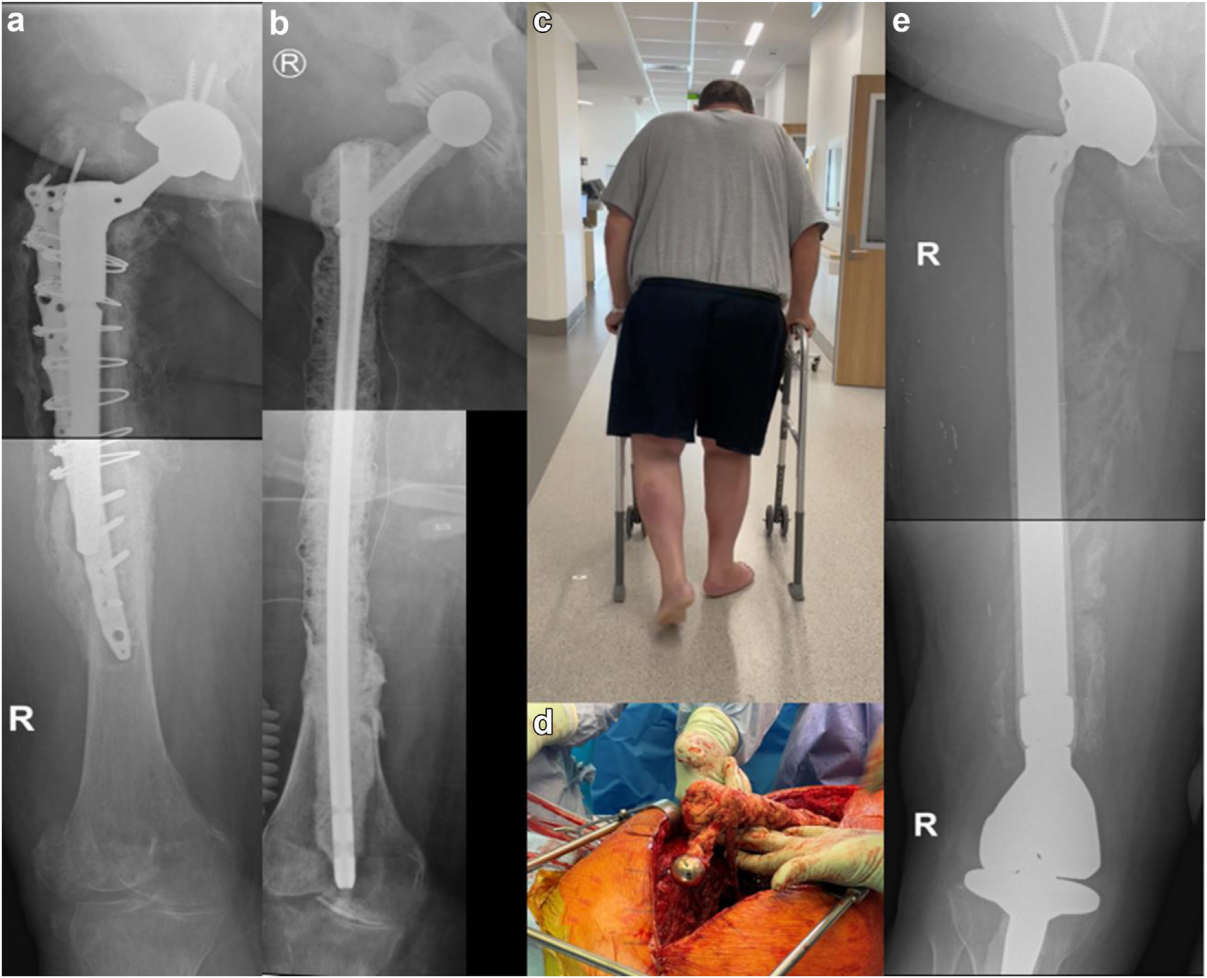
Case 2 RAH. (a) AP radiographs demonstrating the failed revision right THA with extensive osteomyelitis of the femur extending distal to the plate used to treat a periprosthetic fracture. (b) AP radiographs after the first-stage reconstruction of the femur using an antibiotic-cement-coated gamma nail as a temporary megaprosthesis. (c) The patient ambulating with a frame 1 week after his first-stage revision. (d) Clinical intraoperative images of interval prosthesis after hip dislocation at the second-stage revision. (e) AP radiographs of the reconstruction at the second-stage revision with a total femoral replacement prosthesis.

**Figure 4:**
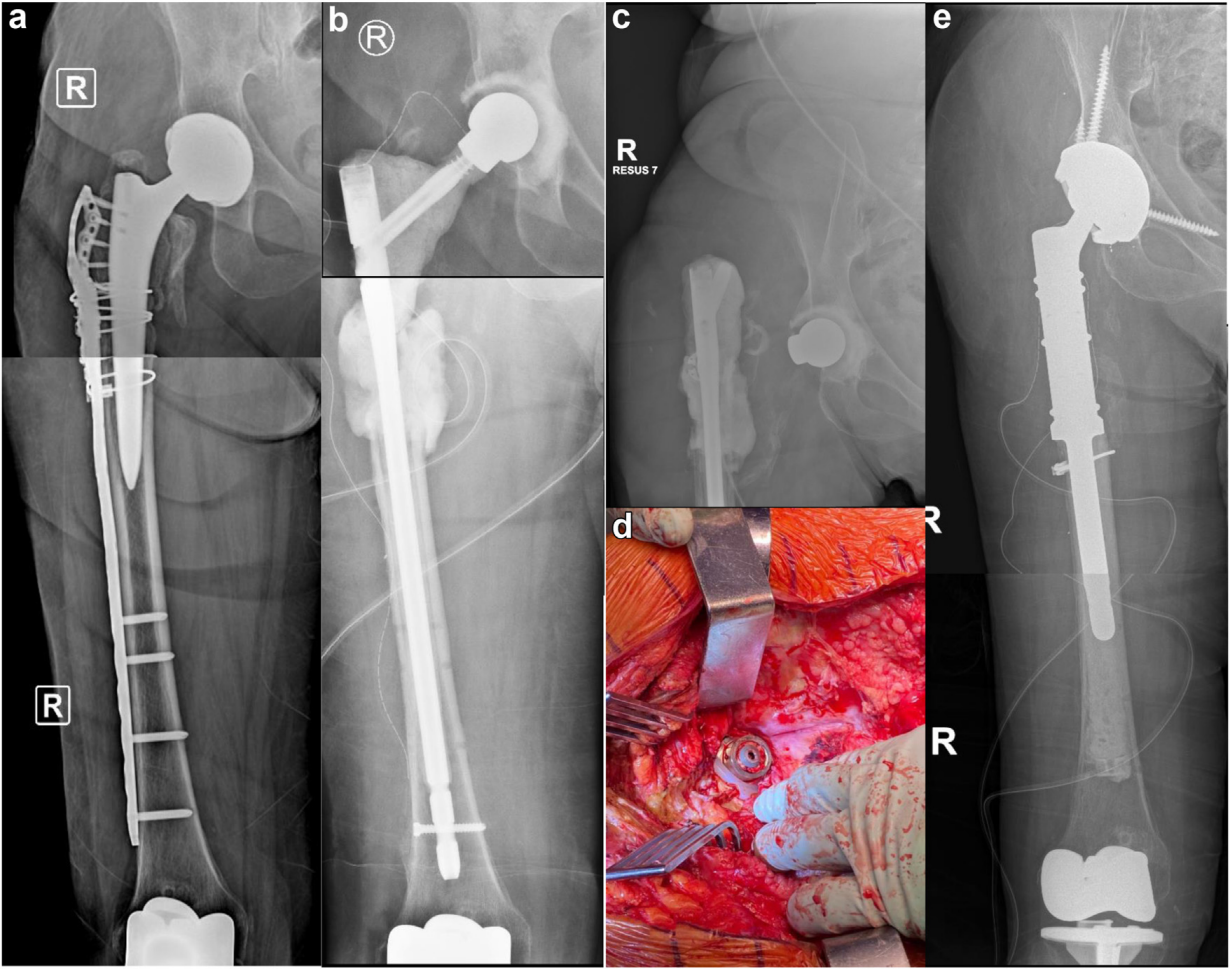
Case 3 RAH. (a) AP radiographs demonstrating infected right interprosthetic plate with nonunion of the lesser trochanter. (b) AP radiograph after the first-stage reconstruction of the femur using an antibiotic-coated-gamma nail as a temporary prosthesis. (c) AP radiograph demonstrating fractured implant prior to second-stage revision. (d) Intraoperative image shows the retained screw threads in the femoral head. Note the absence of metallosis. (e) AP radiograph of second-stage revision to proximal femur replacement prosthesis.

**Figure 5:**
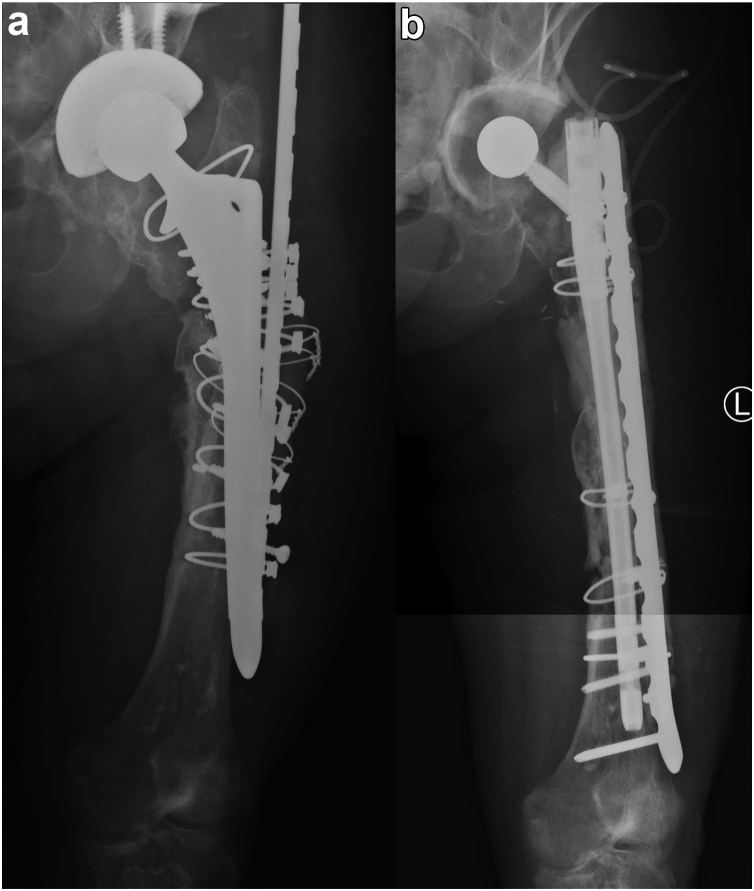
Case 2 QMH (a) Anteroposterior (AP)s X-rays before the first stage revision. (b) AP radiographs after the first stage reconstruction of the femur using a gamma nail and lateral femoral plate coated with antibiotics-loaded cement.

Five cases were reviewed (Ethics #R20010103a; 3 at RAH and 2 at QMH) (Table 1). The first case, presented above, was of a 66-year-old woman infected with methicillin-sensitive Staphylococcus aureus, E. coli, and eventually Pseudomonas aeruginosa and Candida albicans. The gamma nail spacer was converted to a PFR prosthesis coated with antibiotic-loaded cement after 4 weeks, 2 weeks later than initially planned, as she tolerated the implant very well (Fig. 1). The second case, a 59-year-old obese male (143 kg, body mass index – 40.9 kg/m^2^) presented with a multidrug-resistant polymicrobial PJI in February 2021 after a failed staged revision for PJI and a periprosthetic fracture. The patient was wheelchair-bound for 1 year before his first-stage revision with the antibiotic-coated gamma nail. After the first-stage revision, he commenced weight bearing as tolerated with a walking frame (Fig. 3). He underwent a second-stage revision to a total femur replacement 3 months later. The third case, a 75-year-old female nursing home resident presenting with PJI secondary to an interprosthetic fracture fixation presented in September 2022. The second-stage revision was planned for 3 months but was then postponed to 5 months by the patient, as the initially planned surgery was at the beginning of December and the patient did not want to convalesce during the festive season. The lag screw of the gamma nail fractured 5 days before her planned second stage revision when she was admitted and had her surgery brought forward (Fig. 4). The fourth case, first at QMH, was of a 74-year-old female, immunocompromised patient with systemic lupus presenting with a failed revision THA due to PJI. She had a first-stage revision on May 22. Postoperatively, she progressed well to ambulating with a quadpod and refused the second stage. She was last reviewed on February 23, when she declined progression to a second stage (Fig. 2). The fifth case was a 71-year-old female immunocompromised with systemic lupus who developed a PJI with staphylococcus epidermidis after internal fixation of a Vancouver B3 fracture. She underwent a first-stage revision on July 22, after which she continued to ambulate with a frame. This patient was last seen on February 23, when she also refused progression to second-stage revision (Fig. 5).

**Table 1:** Patient and surgical details of 5 cases.

Sl no	Age in y/sex	Comorbidities	Height (cms)/weight (kgs)/BMI	Microbial cultures	Revision to the temporary spacer/cement used/femoral head used	Outcome
1	66/F	Rheumatoid arthritis (RA) on immunosuppression, clostridium difficile colitis	160/83/32.4	Methicillin sensitive *Staphylococcus aureus* (MSSA), *E coli*, *Pseudomonas aeruginosa* and *Candida albicans*	March 2021/ Palacos with premixed 0.5 gm gentamicin to which vancomycin 0.5 gm/pack + voriconazole 0.4 gm/ pack were added – total 4 packs/ Zimmer CoCr METAL HEAD,32 mm, +0, 12/14 taper	Converted to cemented PFR. Ambulating well
2	59/M	Type 2 diabetes mellitus, obesity, hypertension (HT), coronary artery disease with atrial fibrillation, nonalcoholic fatty liver disease (NAFLD).	181/134/40.9	*Lactobacillus salivarius,Pseudomonas aeruginosa*, *Enterococcus raffinosus*, amp-C producing *E coli*, *Klebsiella aerogenes*, Multi-drug resistant (MDR) *Pseudomonas aeruginosa*, *Candida parapsilosis* and *Candida lusitaniae*.	July 2022/ Palacos with premixed 0.5 gm gentamicin to which gentamicin 0.5 gm/pack was added - total 6 packs/ Zimmer CoCr METAL HEAD, 32 mm, +0, 12/14 taper	Oct 2022, Revised to total femur replacement.
3	75/F	Chronic obstructive pulmonary disease, HT, RA, h/o bowel and breast Ca	149/55/24.8	MSSA	Sep 2022/ Simplex P with premixed 1 gm tobramycin to which vancomycin 0.5 gm/pack + amikacin 0.92 gm/pack was added - total 6 packs/ DePuy METAL HEAD, 32 mm, +9	Jan 2023, Revised to proximal femur replacement.
4	74/F	Systemic lupus erythematosis (SLE), HT, osteoporosis	161/72/27.8	*Staphylococcus lugdunensis*	May 2022/ Simplex P with premixed 1 gm tobramycin to which tobramycin 1.2 gm/pack + vancomycin 2 gm/pack was added – total 4 packs/ Stryker METAL HEAD, ,36MM, −4 mm, V40 taper	Declined second stage, Reviewed Feb 2023
5	71/F	SLE	145/50/23.8	*Staphylococcus epidermidis*	July 22/ Simplex P with premixed 1gm tobramycin to which tobramycin 1.2 gm/pack + vancomycin 2gm/pack was added – total 4 packs/ Stryker METAL HEAD, ,36MM, −4 mm, V40 taper	Declined second stage, Reviewed Feb 2023

BMI, body mass index.

## Discussion

These examples suggest that a long gamma nail can be a cheap and effective interval PFR prosthesis at first-stage revision surgery for infection in cases that underwent proximal femoral resection. Other reports described the use of coated femoral nails with antibiotic-loaded cement as interval prosthesis at first revision hip replacement [[6], [7], [8], [9], [10]]. Sanz-Ruiz et al. used a cephalomedullary nail, whereas the other authors used a recon-type nail with 2 screws [[6], [7], [8]]. However, in all these reports, the interval prosthesis was either a hemiarthroplasty, as the proximal screws were used to reinforce a cement prefabricated femoral head construct to articulate directly with the residual acetabular bone, or another high friction arthroplasty where the cemented femoral head articulated with a cement-molded acetabular component. In our technique, the 10.5 mm lag screw of the gamma nail accommodates well a 12/14 taper metal femoral head, thus allowing a low-friction THA articulation. This is important not only to allow the patient a quicker recovery and improved mobility between the stage 1 and stage 2 revisions but also to decrease the overall costs of management of hip PJI through stage revision surgery. Kurtz et al. [11] showed the mean total cost for each stage of a 2-staged revision, where a first stage revision THA vs a second stage revision THA was $35,183 and $31,661 (reported in 2020-adjusted USD), respectively, indicating higher costs toward the first stage in a 2-staged revision THA. In a first stage THA revision, a temporary cement spacer implant is utilized, compared to the more permanent THA prosthesis in the second stage. Hence, the higher cost of the first stage is likely influenced more by a longer length of stay, median 6 days vs 3 days for first stage vs second stage, respectively, rather than prosthesis costs. Kurtz et al. [11] indicated that length of stay was the most significant influencing factor on the total cost of the procedure.

The savings of using a gamma nail instead of a PFR prosthesis were substantial. The cost of a long gamma nail, lag screw, and femoral head was AU$2400 (US$1500). The additional plate screws and cables at QMH brought up the price to AU$8,000 (US5100), which is still substantially cheaper than the price of a PFR prosthesis, which in our institutions ranges between AU$20,000 (US$12800) and AU$40,000 (US$25800) depending on the prosthesis length and manufacturer.

There was no metallosis seen in any patients. The case where the lag screw broke demonstrates that metal fatigue is a risk, like in a trochanteric fracture treated with a gamma nail that progresses to a nonunion [12]. We presume that increasing this patient offset may have improved stability for the patient but could have increased the torque on the implant. Patients should be warned of this potential complication and the fact that they might require a second-stage revision even if they feel satisfied with the result of the first stage. However, it is encouraging to see that the nail could withstand a 134-kg man ambulating unrestricted with a frame for 3 months, as most second-stage revisions are scheduled within this time frame. In conclusion, an antibiotic-coated gamma nail with a metal femoral head articulation in a cemented polyethylene cup is a novel and cost-effective alternative in first-stage surgery to treat PJI.

## Summary

PJI is managed by two staged revisions. Hip PJI management with femoral bone loss is complex and expensive when managed with proximal femur replacement. In this circumstance, we have described a novel surgical technique of antibiotic-coated cephalomedullary nails as a cheap and reliable interval prosthesis before second-stage reconstruction.

## Funding

Stuart Callary held a research fellowship funded by 10.13039/100009727The Hospital Research Foundation Group during this study.

## Conflicts of interest

B. Ramasamy is a faculty of AO Trauma. L. B. Solomon is a faculty of AO Recon; all other authors declare no potential conflicts of interest.

For full disclosure statements refer to https://doi.org/10.1016/j.artd.2023.101218.

## Appendix A Supplementary data

Conflict of Interest Statement for Hammat

Conflict of Interest Statement for Ramasamy

Conflict of Interest Statement for Callary

Conflict of Interest Statement for Solomon

Conflict of Interest Statement for Nelson

Conflict of Interest Statement for Chan
